# Tooth Detection and Numbering Performance of Two AI-Based Systems on Panoramic Radiographs: A Comparative Study

**DOI:** 10.3390/dj14090569

**Published:** 2026-09-06

**Authors:** Sara M. ElKhateeb, Eman Allam, Heba H. Bakhsh, Dur Alomair, Nada Ahmed AlShehri, Nozha Sawan

**Affiliations:** 1 Department of Basic Dental Sciences, College of Dentistry, Princess Nourah bint Abdulrahman University, P.O. Box 84428, Riyadh 11671, Saudi Arabia; 2 Section of Public & Population Health, University of California Los Angeles School of Dentistry, Los Angeles, CA 90095, USA; 3 Department of Preventive Dental Sciences, College of Dentistry, Princess Nourah bint Abdulrahman University, P.O. Box 84428, Riyadh 11671, Saudi Arabia

**Keywords:** artificial intelligence, panoramic radiography, tooth detection, tooth detection and numbering performance

## Abstract

**Objectives:** To evaluate and compare the tooth detection and numbering performance of two commercially available artificial intelligence (AI) software systems, ThakaaMed and EM2AI, in the automated detection and numbering of permanent teeth on panoramic radiographs. **Methods:** A retrospective study was conducted using a randomized sample of 595 panoramic radiographs obtained from multiple dental outpatient facilities. The sample included patients aged 6–18 years with mixed or permanent dentition. Ground truth (GT) annotations were established independently by three experienced dental clinicians using the FDI tooth numbering system. Each radiograph was analyzed using ThakaaMed Dental IQ (Version 1.9) and EM2AI (Version 3.2.0), and AI-generated outputs were compared with GT annotations. Tooth detection and numbering performance was assessed using sensitivity, specificity, and accuracy, and sensitivities and specificities were compared between the two AI systems across anatomical regions. Third molars were analyzed separately because of their variable developmental stages in mixed dentition radiographs. **Results:** EM2AI demonstrated superior performance in third molar detection compared with ThakaaMed. Excluding third molars, overall diagnostic accuracy was 96.81% for ThakaaMed and 99.48% for EM2AI. Regional analyses demonstrated significantly higher sensitivity for EM2AI in the upper anterior, lower anterior, and lower posterior regions, whereas no significant differences in specificity were observed between the two AI systems. EM2AI maintained consistently high tooth detection and numbering performance across anatomical regions. **Conclusions:** Both ThakaaMed and EM2AI demonstrated favorable tooth detection and numbering performance and potential utility as supportive tools in routine dental diagnostic and documentation workflows. EM2AI demonstrated greater consistency across anatomical regions and superior performance in third molar detection. These findings highlight the importance of evaluating AI systems across diverse anatomical regions and developmental stages when assessing their clinical applicability.

## 1. Introduction

Artificial intelligence (AI) has rapidly emerged as a transformative technology in medical and dental imaging. Advances in machine learning and deep learning algorithms have enabled automated interpretation of radiographic images, offering the potential to assist clinicians in diagnosis, improve efficiency, and enhance standardization in clinical decision-making. In dentistry, AI-based systems are increasingly being introduced and applied to various tasks such as caries detection, periodontal bone loss assessment, implant planning, and automated tooth identification on radiographic images [1,2,3].

Tooth detection and numbering represent an important step in many dental diagnostic and treatment planning workflows. Accurate identification of teeth on radiographs is essential for clinical documentation, orthodontic planning, surgical procedures, and digital record management. Early identification of permanent teeth during the mixed dentition stage is essential for timely interceptive treatment planning, including selected extractions in patients with congenitally missing teeth and prevention of eruption disturbances or impactions associated with supernumerary teeth and hypodontia [4,5]. Automated tooth detection systems can streamline these processes by reducing manual workload and improving consistency in radiographic interpretation [5,6]. Therefore, several AI-based dental imaging software platforms have recently incorporated automated tooth detection algorithms.

Despite the growing integration of AI into dental practice, the diagnostic performance of different AI systems may vary depending on algorithm design, training datasets, and quality of the radiographic images [7,8]. Variability in performance may be considerably evident in anatomically complex regions and in cases with developmental variations, such as mixed dentition or partially erupted teeth. Direct comparisons between commercially available AI systems remain limited, and there is a need for independent validation of their diagnostic accuracy under different conditions. The aim of the present study was to evaluate and compare the tooth detection and numbering performance of two AI software systems, ThakaaMed and EM2AI, in detecting permanent teeth on dental panoramic radiographs. The diagnostic outputs of both systems were compared with a ground truth (GT) reference at the individual tooth level and across different anatomical regions.

## 2. Methods

A retrospective diagnostic accuracy study was conducted to evaluate and compare the performance of two commercially available AI software systems, ThakaaMed (AIv4 system, Dental IQ Version 1.9, Riyadh, Saudi Arabia) and EM2AI (EM2AI Version 3.2.0, Clementi, Singapore), in detecting the presence and numbering of permanent teeth on panoramic radiographs of subjects at the mixed dentition stage. The diagnostic outputs generated by both AI systems were compared against a predefined GT reference.

### 2.1. Dataset and Sample Selection

A randomized sample of panoramic radiographs was obtained from the radiographic databases of multiple dental outpatient facilities. The sample included patients aged 6–18 years with either mixed or permanent dentition to reflect a clinically realistic distribution of patient presentations. All radiographs included in the study were of sufficient diagnostic quality for evaluation. A total of 640 anonymized digital panoramic radiographs were initially obtained. Images with severe distortion or poor quality that rendered individual tooth identification impossible were excluded (*n* = 45). Consequently, 595 diagnostically acceptable images were included for the analysis. Image acquisition was performed using multiple panoramic imaging systems across different centers. Standard exposure parameters routinely recommended for panoramic imaging were used (approximately 60–90 kVp, 4–10 mA, and 10–18 s exposure time), with adjustments made according to patient characteristics and machine specifications.

Each radiograph was evaluated for the presence or absence of individual teeth according to the FDI World Dental Federation notation tooth numbering system. Both present and missing teeth were recorded in order to assess the tooth detection and numbering performance of the AI systems.

Third molars were included in the initial individual tooth-level analysis. However, because the dataset contained mixed dentition cases in which third molars were often in early developmental stages, a separate analysis excluding third molars was also conducted.

### 2.2. Ground Truth Determination

GT annotations were established independently by three experienced dental clinicians who were blinded to the outputs of both AI systems. Prior to image assessment, the reviewers were calibrated regarding the study definitions and application of the FDI two-digit tooth numbering system. Each clinician independently evaluated the radiographs for tooth presence, absence, and numbering. In cases of disagreement, the radiographs were jointly reviewed and discussed until consensus was reached. The consensus annotations constituted the final GT reference standard used for all analyses.

### 2.3. AI Systems Evaluation

Each radiograph was independently analyzed using the two AI software systems evaluated in this study: ThakaaMed and EM2AI. Both systems automatically generated tooth detection outputs indicating the presence or absence of teeth at each tooth position.

ThakaaMed Dental IQ (AIv4 system, Dental IQ Version 1.9), developed in Riyadh, Saudi Arabia, is a commercially available AI-driven dental software designed to enhance diagnostic accuracy and optimize clinical workflows. The software provides rapid analysis of dental radiographs, automated dental charting, and AI-assisted treatment planning. EM2AI (Version 3.2.0; Clementi, Singapore) is a commercially available software-as-a-service (SaaS) platform developed for AI-based dental diagnostics. The platform provides web-based support for tooth detection, annotation, and numbering, including both pathological and non-pathological findings. It also generates automated dental charting and AI-assisted treatment plan suggestions based on radiographic analysis.

The AI-generated outputs were extracted and compared with the GT annotations. For each tooth position, diagnostic outcomes were categorized as: true positive (tooth correctly identified as present); false positive (tooth incorrectly identified as present); true negative (tooth correctly identified as absent); and false negative (tooth incorrectly identified as absent). Tooth detection and numbering performance metrics were calculated for each tooth position as well as for regional groupings of teeth.

### 2.4. Regional Tooth Analysis

To explore differences in tooth detection and numbering performance across anatomical regions, teeth were grouped into four regions (upper anterior, lower anterior, upper posterior, and lower posterior). Third molars were excluded from this regional analysis because of their highly variable developmental stage in mixed dentition radiographs, which could introduce bias in AI detection performance.

### 2.5. Tooth Detection and Numbering Performance

For each AI system, tooth detection and numbering performance was evaluated using the sensitivity, specificity, and accuracy metrics [9]. These metrics were calculated at the individual tooth level and for each anatomical region.

### 2.6. Statistical Analysis

Sample size calculation was performed using G*Power (version 3.1.9.7) assuming a medium effect size (0.30), a two-sided significance level (α) of 0.05, and a statistical power of 80%. The analysis indicated that a minimum of 352 images was required. The final sample of 595 images exceeded this requirement, providing adequate statistical power for the analyses performed. Confidence intervals (CIs) for diagnostic accuracy metrics were calculated using the Clopper–Pearson exact method. Sensitivity and specificity estimates were calculated for each AI system with corresponding 95% CIs. Comparisons between ThakaaMed and EM2AI were performed using paired McNemar tests separately for sensitivity (among tooth positions present according to the GT reference) and specificity (among tooth positions absent according to the GT reference). Differences in sensitivity and specificity between the two AI systems were calculated with corresponding 95% CIs. All statistical analyses were performed using R statistical software (version 4.5.2; R Foundation for Statistical Computing, Vienna, Austria).

## 3. Results

### 3.1. Individual Tooth-Level Performance

#### 3.1.1. ThakaaMed

At the individual tooth level, ThakaaMed demonstrated very high sensitivity across most tooth positions. However, performance varied considerably by tooth type, particularly for the third molars. Although sensitivity for third molars was nearly perfect, specificity was extremely low, substantially reducing overall diagnostic accuracy. This imbalance was primarily attributable to a high number of false-positive detections in regions where third molars were absent.

Several posterior teeth demonstrated excellent tooth detection and numbering performance. For example, teeth 37 and 47 achieved 100% sensitivity, specificity, and accuracy. Outside of the third molars, the lowest performance was observed for tooth 32, which demonstrated an accuracy of 78.99% (95% CI: 75.49–82.20). Overall diagnostic accuracy excluding third molars was 96.81%, with a sensitivity of 96.88% and specificity of 83.33%. Detailed tooth-level diagnostic metrics are presented in Table S1.

#### 3.1.2. EM2AI

EM2AI demonstrated strong overall tooth-level detection performance and superior performance in third molar detection compared with ThakaaMed. Performance for third molars was substantially better balanced than that observed with ThakaaMed, with higher specificity while maintaining high sensitivity. Tooth 28 demonstrated an accuracy of 94.79% (95% CI: 92.69–96.43), while tooth 48 demonstrated an accuracy of 90.59% (95% CI: 87.95–92.81). Tooth 15 also demonstrated high tooth detection and numbering performance, with an accuracy of 98.82% (95% CI: 97.59–99.53). Overall diagnostic accuracy excluding third molars was 99.48%, with a sensitivity of 99.58% and specificity of 79.49%. Complete tooth-level diagnostic metrics are presented in Table S2.

#### 3.1.3. Comparison Between AI Systems at the Individual Tooth Level

Both AI systems demonstrated high sensitivity for detecting tooth presence across most tooth positions. However, their performance diverged markedly in the evaluation of third molars. ThakaaMed exhibited near-perfect sensitivity for third molars but extremely low specificity, resulting in a markedly reduced overall accuracy of 45.76%. In contrast, EM2AI maintained balanced sensitivity and specificity, achieving a substantially higher accuracy of 93.19% for third molars.

### 3.2. Regional Comparison Between AI Systems (Excluding Third Molars)

Because the study sample included mixed dentition cases in which third molars were frequently in early developmental stages, third molars were excluded from the regional analyses. Sensitivity and specificity were compared directly between ThakaaMed and EM2AI across the four anatomical regions using paired McNemar tests.

EM2AI demonstrated significantly higher sensitivity than ThakaaMed in the upper anterior, lower anterior, and lower posterior regions. The largest difference was observed in the lower anterior region, where EM2AI demonstrated a 9.76% higher sensitivity (95% CI: 8.80% to 10.79%; *p* < 0.001). Smaller but statistically significant differences favoring EM2AI were observed in the upper anterior (1.66%; 95% CI: 1.18% to 2.20%; *p* < 0.001) and lower posterior (0.78%; 95% CI: 0.48% to 1.12%; *p* < 0.001) regions. No statistically significant difference in sensitivity was identified in the upper posterior region (difference = 0.08%; 95% CI: −0.18% to 0.36%; *p* = 0.516).

No statistically significant differences in specificity were observed between the two AI systems across any anatomical region (all *p* > 0.05). Although differences in specificity estimates were noted in some regions, the confidence intervals were wide and included the null value, reflecting the relatively small number of absent tooth positions available for comparison. Detailed results are presented in Table 1. Overall, EM2AI demonstrated greater consistency in sensitivity across anatomical regions while maintaining specificity comparable to that of ThakaaMed.

### 3.3. Representative Clinical Examples

**Figure 1:**
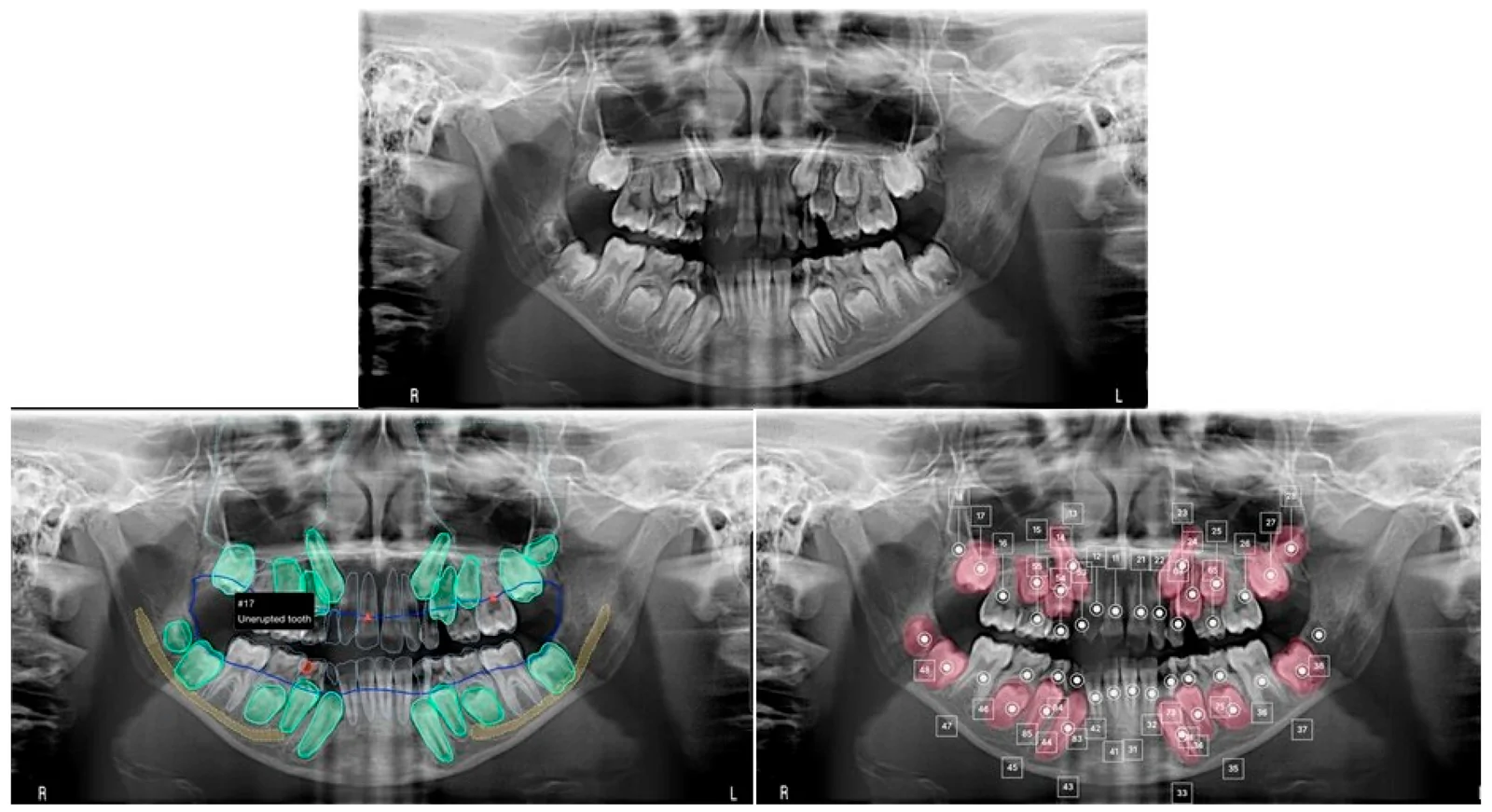
Representative mixed dentition case demonstrating a false-positive tooth detection. The ground truth annotation confirmed the absence of tooth #18. ThakaaMed (right panel) falsely identified tooth #18 as present (false-positive), whereas EM2AI (left panel) correctly identified its absence, showing concordance with the ground truth.

**Figure 2:**
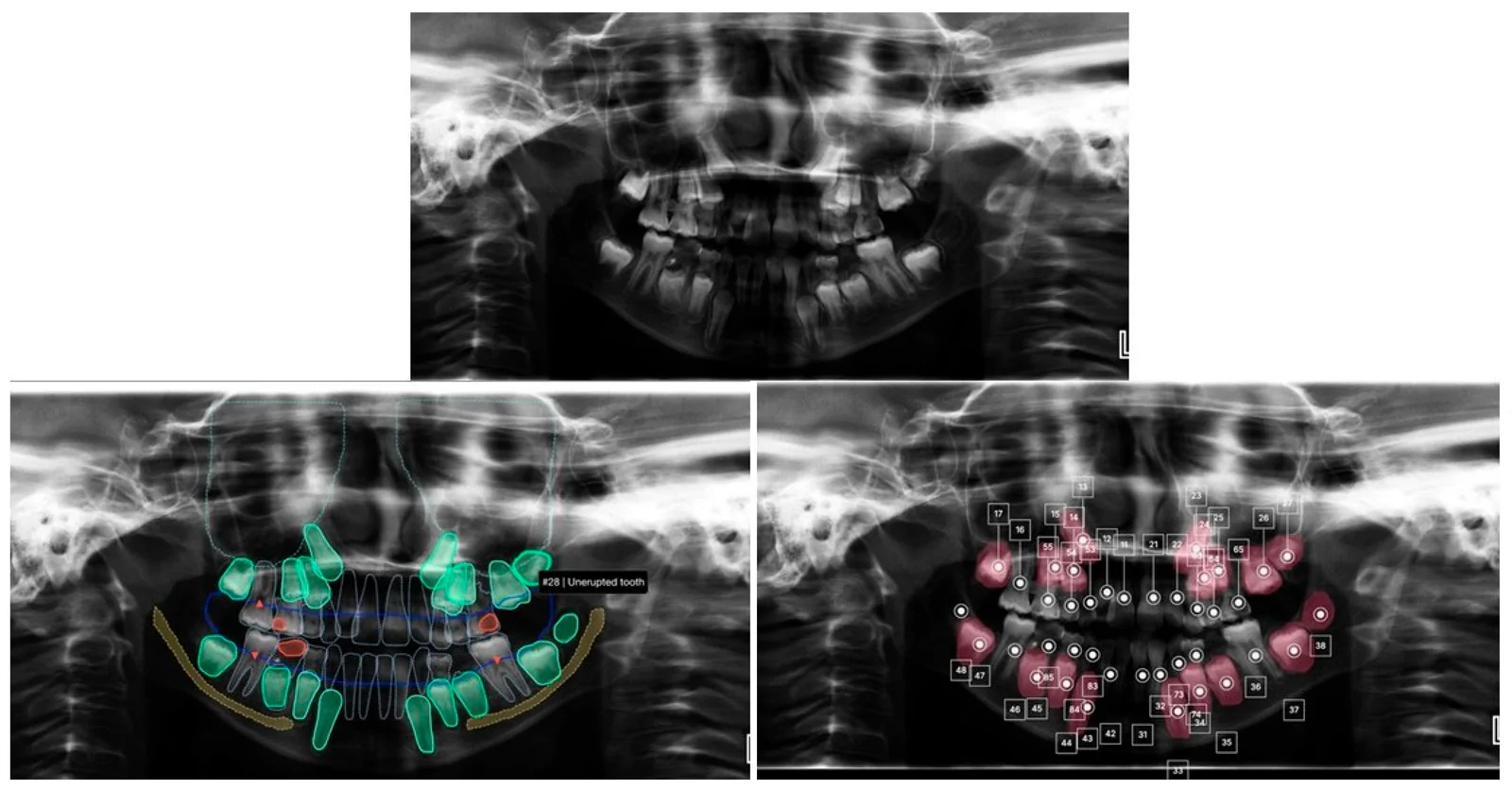
Representative mixed dentition case illustrating a tooth numbering discrepancy. Ground truth confirmed the presence of the unerupted maxillary left second molar (#27). EM2AI (left panel) misclassified this tooth as #28, whereas ThakaaMed (right panel) correctly assigned the tooth number #27, showing complete agreement with the ground truth.

**Figure 3:**
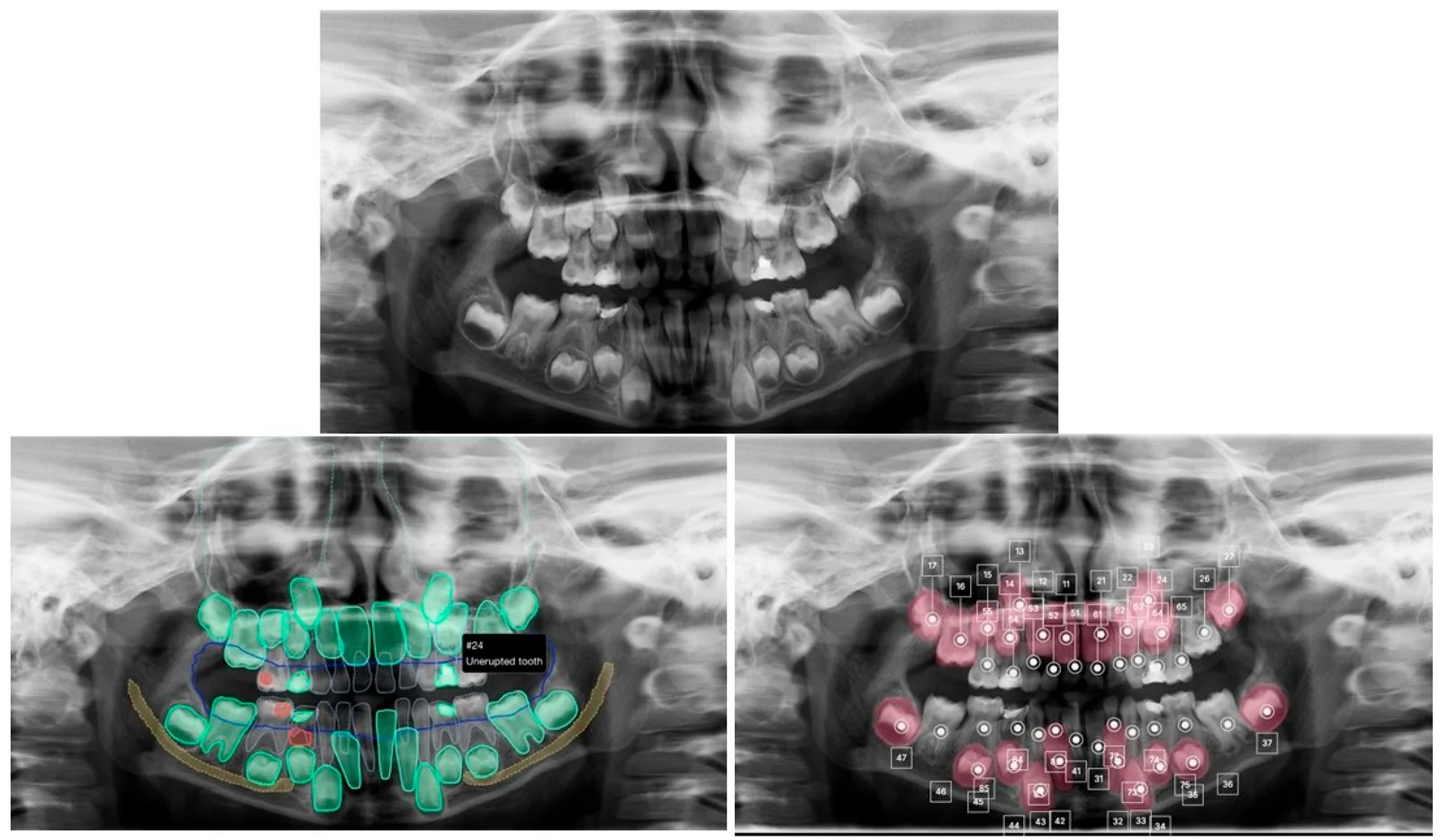
Representative mixed dentition case demonstrating a shared false-negative detection error. The ground truth confirmed the presence of the maxillary left second premolar (tooth #25). However, both EM2AI (left panel) and ThakaaMed (right panel) failed to detect tooth #25, identifying only the adjacent maxillary left first premolar (tooth #24). The missed detection is likely attributable to the lingual eruption path of tooth #25, which caused it to overlap with tooth #24 on the panoramic radiograph, making its identification more challenging. In contrast, the corresponding contralateral tooth on the right side exhibited a normal eruption position and was correctly detected by both AI systems.

**Figure 4:**
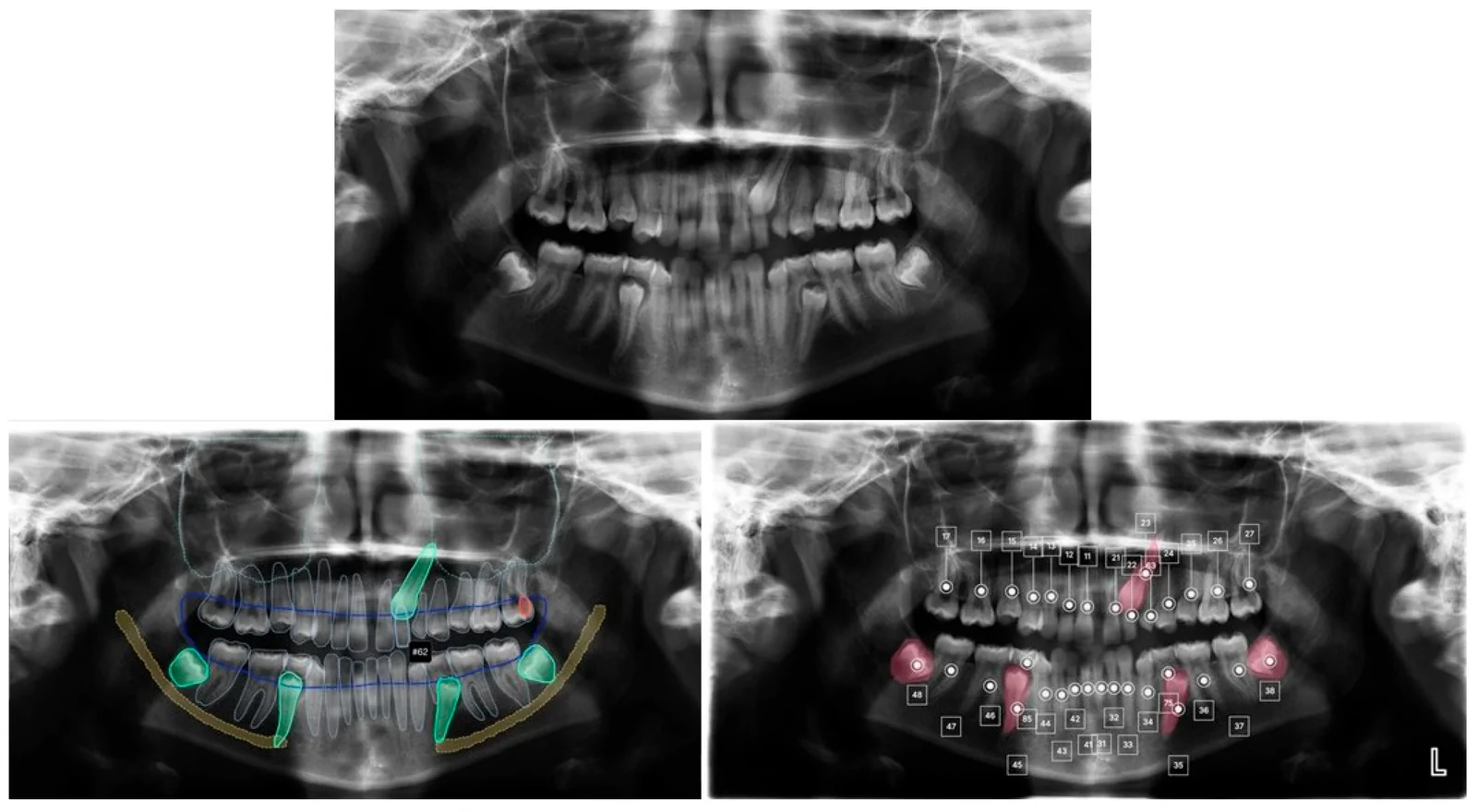
Representative case illustrating misclassification of a retained primary tooth. Ground truth confirmed congenital absence of the maxillary left permanent lateral incisor (#22) and retention of the primary lateral incisor (#62). EM2AI correctly identified the retained deciduous tooth as #62, whereas ThakaaMed misclassified it as the permanent lateral incisor (#22), resulting in an incorrect tooth designation despite successful tooth detection.

To illustrate the performance of both AI systems in clinically relevant scenarios, representative anonymized panoramic radiographs are presented in Figure 1, Figure 2, Figure 3 and Figure 4. These examples demonstrate false-positive tooth detection, tooth numbering discrepancies, shared false-negative detection errors, and misclassification of retained primary teeth in mixed dentition cases.

## 4. Discussion

In digital dentistry, reliable automated tooth detection has several important applications. AI-based systems can assist clinicians in radiographic interpretation, improve workflow efficiency, and support automated charting and treatment planning. In orthodontics, oral surgery, and general dentistry, accurate tooth identification is an essential first step for many AI-driven diagnostic tasks [10,11,12]. Systems that demonstrate consistent performance across anatomical regions may therefore provide greater clinical utility in routine practice. The aim of the current study was to assess and compare the tooth detection and numbering performance of two commercially available AI software systems, ThakaaMed and EM2AI, in detecting the presence and numbering of permanent teeth on panoramic radiographs. The findings indicated that, in their current versions, both systems demonstrated high overall diagnostic accuracy. However, differences were observed in performance consistency across individual teeth and anatomical regions. EM2AI exhibited more stable performance across tooth positions and showed substantially improved detection of third molars compared with ThakaaMed.

At the individual tooth level, both AI systems demonstrated high sensitivity for detecting tooth presence. The largest differences in performance between the two systems were observed in the detection of third molars. Although ThakaaMed demonstrated nearly perfect sensitivity for third molars, its specificity was markedly low, leading to a high number of false-positive detections in areas where third molars were absent. This imbalance substantially reduced overall accuracy for these teeth. In contrast, EM2AI maintained more balanced sensitivity and specificity for third molars, resulting in significantly higher accuracy for these teeth.

The reduced specificity observed for third molars with ThakaaMed may be explained by several radiographic and developmental factors. Third molars frequently demonstrate high anatomical variability, including differences in eruption status, angulation, and degree of mineralization. In younger populations or mixed dentition samples, third molars may appear as partially developed tooth germs or may be completely absent, making their radiographic interpretation more challenging [13,14,15]. AI algorithms trained on datasets with limited variation in third molar morphology or developmental stages may therefore be more prone to false-positive detections in these regions.

When third molars were excluded from the analysis, both AI systems demonstrated excellent overall tooth detection and numbering performance. EM2AI demonstrated significantly higher sensitivity in three of the four anatomical regions evaluated, while no statistically significant differences in specificity were observed between the two AI systems. These findings suggest that EM2AI may provide more consistent tooth detection performance across anatomical regions without compromising its ability to correctly identify absent teeth. This suggests that both systems are highly effective in detecting erupted teeth, but EM2AI may have an advantage in maintaining consistent performance across different anatomical regions.

Regional analysis further supported these findings. ThakaaMed demonstrated strong performance in posterior regions but showed greater variability in the anterior, especially the mandibular anterior region. This may be explained by the radiographic characteristics of anterior teeth in panoramic imaging, where overlapping structures, narrow interproximal spaces, and reduced crown width can make automated detection more challenging [16,17]. EM2AI, in contrast, maintained consistently high accuracy across both anterior and posterior regions, suggesting more robustness of its detection algorithm.

These findings are consistent with the growing body of recent literature demonstrating the effectiveness of AI-based systems in dental radiographic analysis. Previous studies have reported high accuracy for different AI models in detecting dental structures, numbering teeth, and identifying dental conditions on panoramic radiographs [18,19,20,21,22]. Panoramic radiographs are specifically ideal for automated tooth analysis because all teeth are captured in one image. However, anatomical variability, developmental stages, and image quality can influence AI performance [23,24]. In the current study, we identify third molars as a challenge for automated detection systems due to their variable presence and morphology.

Across many fields, AI in digital dentistry now demonstrates near-expert accuracy for object detection and segmentation, and can reliably indicate common pathologies to specific teeth [9,12,18,25]. This can significantly facilitate streamlined charting and support diagnosis, but clinical implementation still requires attention to dataset quality, edge cases, and external validation before full reliance in daily practice. The current study findings report high accuracy for tooth detection and identification. Excluding third molars, ThakaaMed achieved an accuracy of 96.81%, while EM2AI achieved 99.48%.

Despite the promising findings, several limitations should be considered when interpreting the results of this study. The dataset consisted of panoramic radiographs from a single regional clinical setting, which may limit the generalizability of the findings to other populations. Additionally, the inclusion of mixed dentition cases may have influenced third molar detection performance, as early developmental stages can complicate both human and AI interpretation.

The distribution of tooth presence and absence was highly imbalanced, resulting in substantially more positive than negative observations. Consequently, sensitivity estimates were more precise than specificity estimates, which were associated with wider confidence intervals because of the smaller number of absent tooth positions. The retrospective nature of the dataset limited the availability of certain demographic and clinical variables, precluding detailed characterization of the sample beyond the predefined inclusion criteria. Future research should aim to evaluate AI-based tooth detection systems using larger and more diverse multicenter datasets. Further investigating the training datasets and algorithm design of AI models helps identify factors that influence performance variability across tooth types. Additionally, improvements in AI training for developmental stages of teeth, particularly third molars, may enhance the robustness of automated detection systems.

In conclusion, both ThakaaMed and EM2AI demonstrated high tooth detection and numbering performance for automated tooth detection and numbering on panoramic radiographs. EM2AI demonstrated greater consistency across tooth positions and superior performance in the detection of third molars. These findings support the potential utility of AI-assisted tooth identification as an adjunctive tool for radiographic documentation and workflow optimization while highlighting the importance of evaluating AI systems across diverse anatomical regions and developmental stages. The present findings are limited to tooth detection and numbering and do not imply applicability to other diagnostic tasks or independent clinical decision-making.

## Figures and Tables

**Table 1:** Comparison of sensitivity and specificity between ThakaaMed and EM2AI across anatomical regions (excluding third molars).

Quadrant	Sensitivity				Specificity			
	Estimate (95% CI)		Difference (95% CI)	*p*-Value	Estimate (95% CI)		Difference (95% CI)	*p*-Value
	ThakaaMed	EM2AI			ThakaaMed	EM2AI		
Upper anterior	97.80% (97.27% to 98.26%)	99.46% (99.17% to 99.68%)	−1.66% (−2.20% to −1.18%)	<0.001 *	65.00% (40.78% to 84.61%)	60.00% (36.05% to 80.88%)	5.00% (−8.30% to 18.04%)	0.317
Lower anterior	89.79% (88.75% to 90.76%)	99.55% (99.27% to 99.74%)	−9.76% (−10.79% to −8.80%)	<0.001 *	80.00% (28.36% to 99.49%)	60.00% (14.66% to 94.73%)	20.00% (−39.81% to 66.11%)	0.564
Upper posterior	99.41% (99.15% to 99.61%)	99.49% (99.25% to 99.68%)	−0.08% (−0.36% to 0.18%)	0.516	76.92% (46.19% to 94.96%)	76.92% (46.19% to 94.96%)	0.00% (−26.02% to 26.02%)	1
Lower posterior	98.98% (98.65% to 99.25%)	99.77% (99.58% to 99.88%)	−0.78% (−1.12% to −0.48%)	<0.001 *	95.00% (83.08% to 99.39%)	92.50% (79.61% to 98.43%)	2.50% (−9.30% to 14.84%)	0.564

*: Significant (*p* < 0.05).

## Data Availability

The data presented in this study is available on request from the corresponding author.

## Supplementary Materials

The following supporting information can be downloaded at: https://www.mdpi.com/article/10.3390/dj14090569/s1, Table S1: Tooth-level diagnostic performance of the ThakaaMed AI system; Table S2: Tooth-level diagnostic performance of the EM2AI system.
