# Supplementary material for: Tooth Detection and Numbering Performance of Two AI-Based Systems on Panoramic Radiographs: A Comparative Study
Source: Dent J (Basel). 2026 Sep 6;14(9):569. doi: 10.3390/dj14090569 (PMC13604966; doi:10.3390/dj14090569)
Supplement: Supplementary file 1 [file dentistry-14-00569-s001.zip › dentistry-4417856-supplementary.pdf]

Supplementary materials

**Table S1:** Tooth-level diagnostic performance of the ThakaaMed AI system

| Tooth Number | True positive | False positive | True negative | False negative | Value (95% CI) | | |
|---|---|---|---|---|---|---|---|
| | | | | | Sensitivity | Specificity | Accuracy |
| **18** | 225 | 362 | 8 | 0 | 100.00% (98.37% to 100.00%) | 2.16% (0.94% to 4.22%) | 39.16% (35.22% to 43.21%) |
| **17** | 591 | 0 | 3 | 1 | 99.83% (99.06% to 100.00%) | 100.00% (29.24% to 100.00%) | 99.83% (99.07% to 100.00%) |
| **16** | 591 | 0 | 1 | 3 | 99.49% (98.53% to 99.90%) | 100.00% (2.50% to 100.00%) | 99.50% (98.53% to 99.90%) |
| **15** | 587 | 0 | 2 | 6 | 98.99% (97.81% to 99.63%) | 100.00% (15.81% to 100.00%) | 98.99% (97.82% to 99.63%) |
| **14** | 586 | 1 | 0 | 8 | 98.65% (97.36% to 99.42%) | 0.00% (0.00% to 97.50%) | 98.49% (97.15% to 99.31%) |
| **13** | 594 | 0 | 0 | 1 | 99.83% (99.07% to 100.00%) | NA | 99.83% (99.07% to 100.00%) |
| **12** | 571 | 2 | 6 | 16 | 97.27% (95.61% to 98.43%) | 75.00% (34.91% to 96.81%) | 96.97% (95.26% to 98.20%) |
| **11** | 570 | 0 | 0 | 25 | 95.80% (93.86% to 97.26%) | NA | 95.80% (93.86% to 97.26%) |
| **21** | 574 | 1 | 0 | 20 | 96.63% (94.85% to 97.93%) | 0.00% (0.00% to 97.50%) | 96.47% (94.66% to 97.80%) |
| **22** | 570 | 4 | 7 | 14 | 97.60% (96.01% to 98.68%) | 63.64% (30.79% to 89.07%) | 96.97% (95.26% to 98.20%) |
| **23** | 593 | 0 | 0 | 2 | 99.66% (98.79% to 99.96%) | NA | 99.66% (98.79% to 99.96%) |
| **24** | 590 | 0 | 0 | 5 | 99.16% (98.05% to 99.73%) | NA | 99.16% (98.05% to 99.73%) |
| **25** | 586 | 1 | 3 | 5 | 99.15% (98.04% to 99.72%) | 75.00% (19.41% to 99.37%) | 98.99% (97.82% to 99.63%) |
| **26** | 594 | 1 | 0 | 0 | 100.00% (99.38% to 100.00%) | 0.00% (0.00% to 97.50%) | 99.83% (99.07% to 100.00%) |
| **27** | 594 | 0 | 1 | 0 | 100.00% (99.38% to 100.00%) | 100.00% (2.50% to 100.00%) | 100.00% (99.38% to 100.00%) |
| **28** | 239 | 350 | 5 | 1 | 99.58% (97.70% to 99.99%) | 1.41% (0.46% to 3.26%) | 41.01% (37.03% to 45.08%) |
| **38** | 300 | 290 | 5 | 0 | 100.00% (98.78% to 100.00%) | 1.69% (0.55% to 3.91%) | 51.26% (47.16% to 55.35%) |
| **37** | 593 | 0 | 2 | 0 | 100.00% (99.38% to 100.00%) | 100.00% (15.81% to 100.00%) | 100.00% (99.38% to 100.00%) |
| **36** | 591 | 0 | 3 | 1 | 99.83% (99.06% to 100.00%) | 100.00% (29.24% to 100.00%) | 99.83% (99.07% to 100.00%) |
| **35** | 575 | 1 | 14 | 5 | 99.14% (98.00% to 99.72%) | 93.33% (68.05% to 99.83%) | 98.99% (97.82% to 99.63%) |
| **34** | 563 | 1 | 0 | 31 | 94.78% (92.67% to 96.43%) | 0.00% (0.00% to 97.50%) | 94.62% (92.49% to 96.29%) |

| Tooth Number | True positive | False positive | True negative | False negative | Value (95% CI) | | |
|---|---|---|---|---|---|---|---|
| | | | | | Sensitivity | Specificity | Accuracy |
| **33** | 563 | 0 | 0 | 32 | 94.62%<br>(92.49% to 96.29%) | NA | 94.62%<br>(92.49% to 96.29%) |
| **32** | 469 | 0 | 1 | 125 | 78.96%<br>(75.45% to 82.17%) | 100.00%<br>(2.50% to 100.00%) | 78.99%<br>(75.49% to 82.20%) |
| **31** | 541 | 0 | 1 | 53 | 91.08%<br>(88.49% to 93.25%) | 100.00%<br>(2.50% to 100.00%) | 91.09%<br>(88.51% to 93.26%) |
| **41** | 491 | 0 | 0 | 104 | 82.52%<br>(79.23% to 85.49%) | NA | 82.52%<br>(79.23% to 85.49%) |
| **42** | 550 | 1 | 1 | 43 | 92.75%<br>(90.36% to 94.70%) | 50.00%<br>(1.26% to 98.74%) | 92.61%<br>(90.20% to 94.58%) |
| **43** | 587 | 0 | 1 | 7 | 98.82%<br>(97.59% to 99.52%) | 100.00%<br>(2.50% to 100.00%) | 98.82%<br>(97.59% to 99.53%) |
| **44** | 590 | 0 | 1 | 4 | 99.33%<br>(98.28% to 99.82%) | 100.00%<br>(2.50% to 100.00%) | 99.33%<br>(98.29% to 99.82%) |
| **45** | 576 | 0 | 13 | 6 | 98.97%<br>(97.77% to 99.62%) | 100.00%<br>(75.29% to 100.00%) | 98.99%<br>(97.82% to 99.63%) |
| **46** | 590 | 0 | 4 | 1 | 99.83%<br>(99.06% to 100.00%) | 100.00%<br>(39.76% to 100.00%) | 99.83%<br>(99.07% to 100.00%) |
| **47** | 594 | 0 | 1 | 0 | 100.00%<br>(99.38% to 100.00%) | 100.00%<br>(2.50% to 100.00%) | 100.00%<br>(99.38% to 100.00%) |
| **48** | 302 | 288 | 5 | 0 | 100.00%<br>(98.79% to 100.00%) | 1.71%<br>(0.56% to 3.94%) | 51.60%<br>(47.50% to 55.68%) |
| **Overall excluding third molars** | 16064 | 13 | 65 | 518 | 96.88%<br>(96.60% to 97.14%) | 83.33%<br>(73.19% to 90.82%) | 96.81%<br>(96.53% to 97.07%) |
| **All third molars** | 1066 | 1290 | 23 | 1 | 99.91%<br>(99.48% to 100.00%) | 1.75%<br>(1.11% to 2.62%) | 45.76%<br>(43.74% to 47.78%) |

**Table S2:** Tooth-level diagnostic performance of the EM2AI system.

| Tooth Number | True positive | False positive | True negative | False negative | Value (95% CI) | | |
|---|---|---|---|---|---|---|---|
| | | | | | Sensitivity | Specificity | Accuracy |
| **18** | 206 | 6 | 364 | 19 | 91.56%<br>(87.13% to 94.84%) | 98.38%<br>(96.50% to 99.40%) | 95.80%<br>(93.86% to 97.26%) |
| **17** | 591 | 0 | 3 | 1 | 99.83%<br>(99.06% to 100.00%) | 100.00%<br>(29.24% to 100.00%) | 99.83%<br>(99.07% to 100.00%) |
| **16** | 592 | 0 | 1 | 2 | 99.66%<br>(98.79% to 99.96%) | 100.00%<br>(2.50% to 100.00%) | 99.66%<br>(98.79% to 99.96%) |
| **15** | 586 | 0 | 2 | 7 | 98.82%<br>(97.58% to 99.52%) | 100.00%<br>(15.81% to 100.00%) | 98.82%<br>(97.59% to 99.53%) |

| Tooth Number | True positive | False positive | True negative | False negative | Value (95% CI) | | |
|---|---|---|---|---|---|---|---|
| | | | | | Sensitivity | Specificity | Accuracy |
| **14** | 592 | 1 | 0 | 2 | 99.66% (98.79% to 99.96%) | 0.00% (0.00% to 97.50%) | 99.50% (98.53% to 99.90%) |
| **13** | 591 | 0 | 0 | 4 | 99.33% (98.29% to 99.82%) | NA | 99.33% (98.29% to 99.82%) |
| **12** | 585 | 2 | 6 | 2 | 99.66% (98.77% to 99.96%) | 75.00% (34.91% to 96.81%) | 99.33% (98.29% to 99.82%) |
| **11** | 594 | 0 | 0 | 1 | 99.83% (99.07% to 100.00%) | NA | 99.83% (99.07% to 100.00%) |
| **21** | 593 | 1 | 0 | 1 | 99.83% (99.07% to 100.00%) | 0.00% (0.00% to 97.50%) | 99.66% (98.79% to 99.96%) |
| **22** | 576 | 5 | 6 | 8 | 98.63% (97.32% to 99.41%) | 54.55% (23.38% to 83.25%) | 97.82% (96.29% to 98.83%) |
| **23** | 592 | 0 | 0 | 3 | 99.50% (98.53% to 99.90%) | NA | 99.50% (98.53% to 99.90%) |
| **24** | 589 | 0 | 0 | 6 | 98.99% (97.82% to 99.63%) | NA | 98.99% (97.82% to 99.63%) |
| **25** | 586 | 1 | 3 | 5 | 99.15% (98.04% to 99.72%) | 75.00% (19.41% to 99.37%) | 98.99% (97.82% to 99.63%) |
| **26** | 594 | 1 | 0 | 0 | 100.00% (99.38% to 100.00%) | 0.00% (0.00% to 97.50%) | 99.83% (99.07% to 100.00%) |
| **27** | 593 | 0 | 1 | 1 | 99.83% (99.07% to 100.00%) | 100.00% (2.50% to 100.00%) | 99.83% (99.07% to 100.00%) |
| **28** | 214 | 5 | 350 | 26 | 89.17% (84.53% to 92.80%) | 98.59% (96.74% to 99.54%) | 94.79% (92.69% to 96.43%) |
| **38** | 252 | 2 | 293 | 48 | 84.00% (79.35% to 87.96%) | 99.32% (97.57% to 99.92%) | 91.60% (89.07% to 93.70%) |
| **37** | 591 | 0 | 2 | 2 | 99.66% (98.79% to 99.96%) | 100.00% (15.81% to 100.00%) | 99.66% (98.79% to 99.96%) |
| **36** | 591 | 1 | 2 | 1 | 99.83% (99.06% to 100.00%) | 66.67% (9.43% to 99.16%) | 99.66% (98.79% to 99.96%) |
| **35** | 578 | 1 | 14 | 2 | 99.66% (98.76% to 99.96%) | 93.33% (68.05% to 99.83%) | 99.50% (98.53% to 99.90%) |
| **34** | 592 | 1 | 0 | 2 | 99.66% (98.79% to 99.96%) | 0.00% (0.00% to 97.50%) | 99.50% (98.53% to 99.90%) |
| **33** | 594 | 0 | 0 | 1 | 99.83% (99.07% to 100.00%) | NA | 99.83% (99.07% to 100.00%) |
| **32** | 590 | 0 | 1 | 4 | 99.33% (98.28% to 99.82%) | 100.00% (2.50% to 100.00%) | 99.33% (98.29% to 99.82%) |
| **31** | 591 | 1 | 0 | 3 | 99.49% (98.53% to 99.90%) | 0.00% (0.00% to 97.50%) | 99.33% (98.29% to 99.82%) |
| **41** | 595 | 0 | 0 | 0 | 100.00% (99.38% to 100.00%) | NA | 100.00% (99.38% to 100.00%) |
| **42** | 586 | 0 | 2 | 7 | 98.82% (97.58% to 99.52%) | 100.00% (15.81% to 100.00%) | 98.82% (97.59% to 99.53%) |
| **43** | 593 | 1 | 0 | 1 | 99.83% (99.07% to 100.00%) | 0.00% (0.00% to 97.50%) | 99.66% (98.79% to 99.96%) |

| Tooth Number | True positive | False positive | True negative | False negative | Value (95% CI) | | |
|---|---|---|---|---|---|---|---|
| | | | | | Sensitivity | Specificity | Accuracy |
| **44** | 593 | 0 | 1 | 1 | 99.83% (99.07% to 100.00%) | 100.00% (2.50% to 100.00%) | 99.83% (99.07% to 100.00%) |
| **45** | 581 | 0 | 13 | 1 | 99.83% (99.05% to 100.00%) | 100.00% (75.29% to 100.00%) | 99.83% (99.07% to 100.00%) |
| **46** | 590 | 0 | 4 | 1 | 99.83% (99.06% to 100.00%) | 100.00% (39.76% to 100.00%) | 99.83% (99.07% to 100.00%) |
| **47** | 593 | 0 | 1 | 1 | 99.83% (99.07% to 100.00%) | 100.00% (2.50% to 100.00%) | 99.83% (99.07% to 100.00%) |
| **48** | 249 | 3 | 290 | 53 | 82.45% (77.68% to 86.57%) | 98.98% (97.04% to 99.79%) | 90.59% (87.95% to 92.81%) |
| **Overall excluding third molars** | 16512 | 16 | 62 | 70 | 99.58% (99.47% to 99.67%) | 79.49% (68.84% to 87.80%) | 99.48% (99.36% to 99.59%) |
| **All third molars** | 921 | 16 | 1297 | 146 | 86.32% (84.11% to 88.32%) | 98.78% (98.03% to 99.30%) | 93.19% (92.11% to 94.17%) |
